# Our New York Letter

**Published:** 1891-01

**Authors:** 


### Correspondence.

### OUR NEW YORK LETTER.

NEW YORK, Dec. 18, 1890.

Considerable discussion has been going on in the daily papers here concerning an operation that has been performed on a boy in one of the city hospitals. The operation had in it a sensational element, and, of course, was eagerly seized upon and digested by the newspaper reading portion of the community. It was in the nature of an experimental essay, and one of a character not to be criticized by the daily press, but the opportunity that presented was too good to be lost of getting a free advertisement, and the matter was unfortunately given to the papers for publication. The result has been that no operations of a similar character will again be permitted to be performed in that hospital, and the newspapers are advocating restrictive legislative measures in this direction.

Medical circles have been greatly disturbed about the whole affair, and the surgeon who performed the operation, and who, they think, gave it to the press, has been strongly censured. Apropos of this affair, a well known physician of this city said the other day to the writer: "It is astonishing to note the precipitancy with which doctors in this city rush into print on the slightest provocation; and that too, without any material benefit accruing to themselves. Specialists who depend largely for practice on the good will and esteem of the profession at large, cannot for a moment imagine that by posing as great men in the daily papers, they thereby earn the respect and confidence of their brother practitioners. It is not by that means great and lasting practice is built in this city. Take any of the men who are professionally known and note the large practices they have got, for none of which are they indebted to this system of advertising, such as is afforded in the columns of the daily papers. I regard those men who are constantly parading in the daily press as great specialists, as no better than quacks and humbugs and have little faith in their honesty. By doing this they are violating one of the essential elements of the code, and men who do so unblushingly seem to me to be destitute of all sense of honor."

Dr. Wm. T. Bull, in speaking at a recent clinic in this city, on cancer of the pylorus, said it was the duty of every physician when an old person came to his office suffering from dyspeptic symptoms to examine carefully for cancer of the pylorus, and if on examining the abdomen he detected the presence of a well defined tumor, it was his duty to operate at once; and there was every reason to expect a cure by operative measures in the early stages of the disease.

Dr. Fessenden G. Otis, speaking recently of cancer of the fingers, said that whenever you find a sluggish sore on the finger of a surgeon or accoucheur, you may almost always suspect it to be syphilitic in character. There are certain characteristics about it that when once seen you can never forget. Two years ago he happened to be at Lakewood when he saw a gentleman going about with a handkerchief rolled around his fingers. He came to him (Dr. Otis) and introduced himself as a surgeon who had a large practice in the West. He showed him the finger and asked him what he thought about it. He told Dr. Otis that he performed an ordinary hairlip operation on a young German girl who had cancer of the lip, and in doing so had wounded his finger with the knife. A troublesome sore soon made its appearance about the nail. Recognizing the fact that the sore had something to do with the cancer of the young girl, the finger was laid open, thoroughly scraped down to the bone, dressed antiseptically and put in a plaster bandage. This was the dressing it was put up in when Dr. Otis saw it. The doctor took him to his room and examned him very carefully. He had a very characteristic eruption all over the body, and enlarged glands, in every part with the exception of the epitrochlear. He was put on a mixed treatment and requested to go abroad, as he was unable to do work of any kind. He went to England, and while there he consulted Sir Henry Thompson with regard to his trouble. Sir Henry examined him carefully, noted the eruption and said there was nothing syphilitic about it. He advised him to throw his medicines away and go home. He returned to this country and related the circumstances of his visit to Sir

Henry Thompson to Dr. Otis. He was then advised by Dr. Otis to hunt up the girl on whom he operated for harelip, and he did so. He found she had very severe manifestations of secondary syphilis.

Dr. Beverly Robinson recognizes a very close and intimate relationship between acute tonsilitis and rheumatism. He says he has seen a great many of these cases in his wards at the New York hospital and found the greater majority of them yield very readily to ammoniated tincture of guiaicum or salol. He also believes that suppuration can in many cases be prevented by the use of these remedies which are certainly anti-rheumatic in effect.

Dr. Simon Baruch, of this city, is a great believer in the cold bath treatment of typhoid fever. He says it yields the most triumphant results in combatting the effects of toxic agents. The reflex stimulus aroused by the shock to the peripheral nerve endings so energizes the nerve centres which furnish innervation for circulation, respiration, digestion, tissue-formation and excretion, that the system is enabled to tide over the dangers which would result from failure of these functions. Antipyretics diminish the excretion of urea and nitrogen, and hence diminish the excretion of the materies morbi through the kidneys. The liver in patients dying after treatment by antipyrine is from 6 to 12-50 grammes heavier than in those dying after the cold bath.

Dr. Jacobi is of the opinion that spartein, an alkaloid of scoparins, is a more effective cardiac tonic than digitalis. He says that digitalis from the fact of its slow absorption, and consequent slow elimination, is apt to give rise to cumilative effects, while this is not true of spartein. If you desire to secure stimulating effects very rapidly, you can give a few doses of spartein at once, and it is absorbed immediately and as quickly eliminated.

Dr. A. L. Loomis has formulated rules under which it is safe to give digitalis in cases of heart failure. If in a case of advanced heart failure from dilatation, the urinary secretion is increased by the use of digitalis, it is safe to continue its use as long as the amount of urine exceeds or equals the normal. If, howerer, during its administration, the amount of the urine diminishes, the digitalis should be at once stopped. Should such a warning be neglected, the ventricular contractions will become more and more feeble, until, finally, we get, perhaps, a complete stoppage of the heart. In cardiac dilatation where high, arterial tension exists, the urine will not be increased by digitalis, and its use is contraindicated.

The quantity of digitalis in any given case varies with the effect. If five or ten drops of the tincture three times a day relieve the symptoms of an overdistended ventricle, no larger doses should be given. The best effects of the drug are always obtained when the patient is at rest, and, therefore, the largest dose should be given at night on retiring to bed. Patients who require large doses of the drug to produce an effect should be kept in bed during its administration, and the maximum dose it is safe at any time to give a patient is sixty minims of the tincture or half an ounce of the infusion.

Strophanthus and convallaria, he considers far less reliable than digitalis; while more prompt in their action than digitalis, their effect is less permanent and they have no diuretic aciton.

### MENTHOL FOR CHAPPED HANDS.

A writer in the *Provincial Medical Journal* offers the following: Menthol, 15 grains; salol, 1-2 drachm; olive oil, 1-2 drachm; lanolin, 1 1-2 ounces, as a soothing application for chapped hands. The pain, he says, is at once allayed after the first application, and the skin at the same time is softened. The fissures will heal promptly under a systematic use of the application once or twice daily.—*N. J. Med. Jour.*

### SUICIDES IN FRANCE.

In France, from 1827 to 1880, about two hundred thousand persons committed suicide. Of these, over fifteen thousand men and eleven hundred women women were inebriates, and intoxicated at the time of death.—Ex.

